# Phase angle and Mediterranean diet in patients with acne: Two easy tools for assessing the clinical severity of disease

**DOI:** 10.1186/s12967-021-02826-1

**Published:** 2021-04-26

**Authors:** Luigi Barrea, Marianna Donnarumma, Sara Cacciapuoti, Giovanna Muscogiuri, Ludovica De Gregorio, Chiara Blasio, Silvia Savastano, Annamaria Colao, Gabriella Fabbrocini

**Affiliations:** 1 Dipartimento di Scienze Umanistiche, Centro Direzionale, Università Telematica Pegaso, Via Porzio, isola F2, 80143 Napoli, Italy; 2 Endocrinology Unit, Department of Clinical Medicine and Surgery, Centro Italiano per la cura e il Benessere del paziente con Obesità (C.I.B.O), University Medical School of Naples, Via Sergio Pansini 5, 80131 Naples, Italy; 3 Section of Dermatology, Department of Clinical Medicine and Surgery, University of Naples Federico II, Naples, Italy; 4 Unit of Endocrinology, Dipartimento di Medicina Clinica e Chirurgia, Federico II University Medical School of Naples, Via Sergio Pansini 5, 80131 Naples, Italy; 5 Cattedra Unesco “Educazione alla salute e allo sviluppo sostenibile”, University Federico II, Naples, Italy

**Keywords:** Acne, Phase angle, Body composition, Bioelectrical impedance analysis (BIA), Mediterranean diet, Nutrition, Nutritionist

## Abstract

**Background:**

Acne is a chronic, inflammatory and debilitating skin disorder. Dietary factors and nutritional status are among the exacerbating factors of acne. Phase angle (PhA), a direct measure of Bioelectrical Impedance Analysis (BIA), represents an indicator of the chronic inflammatory state. The Mediterranean diet (MD) is a healthy dietary pattern that can exert anti-inflammatory effects in several inflammatory diseases. We aimed to investigate the difference in PhA and adherence to the MD and their associations with the severity of acne in a sample of naïve treatment patients with acne compared to control group.

**Materials:**

In this cross-sectional, case–control, observational study, we enrolled 51 patients with acne and 51 control individuals. Body composition was evaluated by a BIA phase-sensitive system (50 kHz BIA 101 RJL, Akern Bioresearch, Florence, Italy, Akern). For adherence to the MD, we have used the PREvención con DIeta MEDiterránea (PREDIMED) questionnaire. The clinical severity of acne was assessed by using the global acne grading system (GAGS), a quantitative scoring system to assess acne severity.

**Results:**

Patients with acne had a worse body composition, in particular smaller PhA (*p* = 0.003), and a lower adherence to the MD (*p* < 0.001) than the control group, in spite of no differences in gender, age and BMI between the two groups. Stratifying patients with acne according to GAGS categories, both PhA (*p* = 0.006) and PREDIMED score (*p* = 0.007) decreased significantly in severe acne than mild/moderate acne. The GAGS score was negative correlations with PhA (r = − 0.478, *p* < 0.001) and PREDIMED score (r = − 0.504, *p* < 0.001). The results of the multivariate analysis showed PhA and PREDIMED score were the major determinants of GAGS score (*p* < 0.001). The receiver operator characteristic (ROC) analysis reporting a value of PhA of ≤ 6.1° and a PREDIMED score of ≤ 9 identified patients with acne with the highest clinical severity of the disease.

**Conclusions:**

Novel correlations were reported between PhA and the degree of adherence to the MD with acne severity. Of interest, PhA and PREDIMED scores might represent possible markers of the severity of acne in a clinical setting. This study highlights how a cooperation between dermatologist and nutritionists might provide a combination key in the complex management of acne patients.

## Background

Acne is a complex and a chronic inflammatory cutaneous disorder involving the pilosebaceous unit and its multifactorial pathogenesis is attributed to multiple factors such as hyperseborrhea, hyperkeratinization of the pilosebaceous duct, colonization of Propionibacterium acnes and perifollicular inflammation [1]. The influence of skin and gut microbioma in acne pathogenesis has also been investigated [2]. Its prevalence varies in overtime and different countries, and different lifestyles may influence it [3, 4]. This skin disorder affects 70–80% of adolescents, persisting into the 20 s and 30 s in roughly 64 and 43% of individuals, respectively [5, 6]. Acne can leave lifelong scars and hyperpigmentations that need to be correctly prevented and treated [7, 8] considering that they impact considerably on patients’ quality of life and emotional health [9]. The lesional pleomorphism is a typical aspect of patients with acne, represented by contemporary different lesions in the same patient, which can be both inflammatory lesions (papules, pustules, and nodules) and noninflammatory lesions such as comedones. In this context, acne may be present in a wide variety of clinical manifestations depending on the severity of the predominant lesion, number, and type [1]. The abnormal desquamation of sebaceous follicle epithelium (comedogenesis), sebaceous gland hyperplasia with seborrhea, increased bacterial colonization of the follicle (*Propionibacterium acnes*), and immunological and inflammatory factors, are the most notable pathophysiological factors that influence the development of acne are [10]. The immunochemical pathways linked to the state of inflammation in acne are complex and involves several inflammatory mediators and their target receptors, including cytokines, defensins, peptidases, sebum lipids, and neuropeptides [11]. In particular increased prostaglandin E2 and PPARγ levels can induce sebaceous gland hyperplasia and overshooting sebum production [12]. These sebum changes might induce inflammation leading to acne lesions. In addition, also *Propionibacterium acnes* has been shown to trigger pro-inflammatory cytokine release [12].

Beyond genetic factors, considered as the main cause in the development of acne, several evidence reported that commonly attribute the acne condition or its exacerbation also to hormonal influences and dietary factors [13, 14]. Despite the scientific literature reporting contradictory result and conclusions on the role of diet in acne, due especially to the numerous limitations of study design, recently some well-designed, controlled, prospective studies have shown the relationship among specific nutrients and clinical severity of acne [13, 15–17]. Of interest, associations have been reported between cow’s milk intake and consumption of high-glycaemic index foods with presence and longer acne duration [17]. Mediterranean diet (MD) is characterized by high consumption of extra virgin olive oil, fish, vegetables, legumes, whole-grain products, fruits, and nuts. Therefore, MD appears to be a low-glycaemic index diet and for this reason, it could be evaluated as a protective factor against acne development [17]. Of interest, MD as lifestyle, shows several benefits on different clinical inflammatory settings, including psoriasis [18, 19], hidradenitis suppurativa [20], polycystic ovary syndrome [21, 22], breast cancer [23–25], menopause [26, 27], and in all inflammatory and immune processes [28, 29]. MD decreases inflammation and oxidative stress, providing a significant source of antioxidant vitamins [30]. Nevertheless, to date, only few evidence reported the role of the MD on the development or exacerbation of acne [31].

In clinical practice, in addition to the evaluation of eating habits, the assessment of body composition has an important role. It is commonly measured by bioelectrical impedance analysis (BIA), a noninvasive, simple, and inexpensive method for the evaluation of body composition. BIA-device has a high agreement with Dual-energy X-ray absorptiometry, the gold standard in the evaluation of body composition [32, 33]. Phase angle (PhA) is a BIA-derived measure and that is associated with the inflammatory status in several diseases [34, 35]. In both healthy subjects and individuals with obesity, PhA has been validated as an easy tool to detect the inflammatory process [36–38]. PhA represents an excellent indicator of physical state, and cellular integrity, as well as of the water distribution between the extracellular (ECW) and intracellular water (ICW) compartments [39, 40]. Of interest, the PhA is an important prognostic index for monitoring the presence and evolution of chronic inflammatory processes [41]. Nevertheless, to the best of our knowledge, no studies to date have assessed PhA as a potential inflammatory marker in patients with acne.

Thus, considering the existence of inflammation in patients with acne, and while taking into account the well-known anti-inflammatory effects of MD, we suppose that patients with acne should be more prone to follow an unhealthy nutritional patter, lowly adherent to the MD, which contributes to worsening both inflammation and, consequently, the clinical severity of acne. Furthermore, since several studies demonstrated the role of PhA, as an inflammatory state, we hypothesize to find this association also in patients with acne.

In this context, the main aim of the present study was to evaluate adherence to the MD and the body composition assessed by BIA and their association with the presence of acne and its clinical severity in a cohort of treatment-naïve patients with acne as compared with a control group of healthy individuals matched for gender, age, and BMI.

## Material and methods

### Design and setting

This cross-sectional, case–control, observational study was carried out in patients with acne attending the Unit of Dermatology, Department of Clinical Medicine and Surgery, University Federico II of Naples (Italy), from January 2019 to February 2020. This study was carried out in accordance with the Code of Ethics of the World Medical Association (Declaration of Helsinki) for experiments involving humans, which has been approved by the Local Ethical Committee (no. 05/14). To the study participants, it was clearly explained the aim of this study and a written informed consent was obtained.

### Population study

The study included 51 treatment-naïve patients affected by acne attending the Outpatient Clinic of the Unit of Dermatology in our Department. Fifty-one Caucasian healthy subjects (ascertained from medical history by an Endocrinologist), gender, age, and BMI matched were chosen as controls among hospital volunteers, employees from the same geographical area around Naples (Italy), or subjects who participated in the OPERA project (obesity, programs of nutrition, education, research and assessment of the best treatment) a Prevention Project held in Naples on 11–13 October 2019 [42]. The OPERA Prevention Project is also a strategic project of the UNESCO Chair on “Health Education and Sustainable Development” (https://www.unescochairnapoli.it/ for details).

All female subjects were evaluated in the follicular phase of the menstrual cycle and were not pregnant and non-lactating. A full medical history, including drug use, was collected.

Inclusion criteria for all groups were: individuals who were normal weight, overweight or with obesity, aged 18–42 years (women of childbearing age), lack of underlying metabolic disease (type 2 diabetes, hypertension, diagnosed anaemia, or any other metabolic disease requiring a special diet).

To increase the homogeneity of the subject samples, we included only patients with acne treatment-naïve and only adults of both genders with the following criteria of exclusion:

1. Age < 18 years and > 43 years;
2. Subjects with a diagnosis of acne lasting > 6 months or were receiving any systemic treatment for acne including acitretin, ciclosporin, methotrexate, phototherapy or biologics for at least 3 months;
3. For females: menopause (defined as amenorrhea for ≥ 3 years or amenorrhea for ≥ 1 but < 3 years and plasma follicle-stimulating hormone concentrations elevated to the postmenopausal range); pregnancy or lactation in the past 6 months;
4. Subjects with a self-reported history of recent weight change (> 10% weight change within the last 6 months);
5. Endocrine disorders that could affect body composition or nutritional status, such as hyperandrogenism and/or biochemical hyperandrogenaemia, oligomenorrhea due to polycystic ovarian syndrome or secondary aetiologies according to the Endocrine Society, including thyroid dysfunction, Cushing’s syndrome, adrenal disorders, androgen-secreting tumors, congenital adrenal hyperplasia, and hyperprolactinaemia [43], altered thyroid hormone function tests or thyroid hormone treatment, and other chronic diseases that could interfere fluid homeostasis, such as liver or renal chronic diseases, cancer, acute or chronic inflammatory diseases, and presence of type 2 diabetes;
6. Use of medications that impact nutrients metabolism (oral contraceptive pills, metformin, anti-epileptics, anti-psychotics, statins, and fish oil), on body composition (drugs that could influence fluid balance, including nonsteroidal anti-inflammatory drugs, diuretics, laxative use), or weight-loss medications;
7. Hypocaloric diet or specific dietary regimens in the last three months, including vegan or vegetarian diets; supplementation with dietary supplements including antioxidants, vitamins, or minerals;
8. Individuals with implanted pacemakers or defibrillators because of the theoretical possibility of interference with the BIA-device activity due to the field of current induced by the impedance measurements.

### Sample size justification and power

The calculation of the sample size was performed by considering two independent study groups, the effect size 0.95 with a type I error of 0.05 and a power of 95%, as previously reported in other studies [44, 45]. The number of subjects to be enrolled was found to be 50 per group that we decided to round up to 51 with a total of 102 total subjects enrolled in the study to replace drop patients. The power was calculated by the differences of means ± standard deviation (SD) of PREDIMED score in patients and control group (5.59 ± 2.89 vs 10.67 ± 1.78, respectively). Considering the number of cases required in each group of 50, which were set at 51 for each group, a type I (alpha) error of 0.05 (95%), and a type II (beta) of 0.05, the calculated power size was 95%. The calculation of sample size and power were performed using Sample Size Calculator Clinical Calc (https://clincalc.com/stats/samplesize.aspx).

### Lifestyle habits

Were defined as physically active, individuals habitually engaged in at least 30 min/day of aerobic exercise (YES/NO). We defined as former smokers were subjects who stopped smoking at least one year before the interview, current smokers subjects smoking at least one cigarette *per* day, and non-current smokers as the remaining participants; as we have already fully reported in previous studies [46–48]

### Anthropometric measurements

The anthropometric measurements were performed by a certified clinical nutrition specialist with 5 years of experience in dietetics and nutrition, in the morning, between 8 and 10 am, after an overnight fast, according to the International Society for the Advancement of Kinanthropometry (ISAK 2006). All subjects dressed with light clothes without shoes during the assessment, as previously reported [49–52]. A calibrated balance beam scale (Seca 711; Seca, Hamburg, Germany) was used to assess weight, while a wall-mounted stadiometer (Seca 711; Seca, Hamburg, Germany) was used to measure height. After measuring weight and height, the BMI [weight (kg) divided by height squared (m^2^), kg/m^2^] was calculated.

According to World Health Organization WHO’s criteria, we defined the participants as follows: BMI: 18.5–24.9 kg/m^2^, normal-weight; BMI: 25.0–29.9 kg/m^2^, overweight; BMI: 30.0–34.9 kg/m^2^, grade I obesity; BMI: 35.0–39.9 kg/m^2^, grade II obesity; BMI ≥ 40.0 kg/m^2^, grade III obesity [53]. In according to the National Center for Health Statistics [54], using a non-stretchable measuring tape at the natural indentation or at a midway level between the lower edge of the rib cage and the iliac crest if no natural indentation was visible, the waist circumference was measured to the closest 0.1 cm.

### Body composition

Body composition was assessed using a BIA phase-sensitive system by a certified clinical nutrition specialist with 5 years of experience in the assessment of body composition with the BIA-method (800-µA current at a frequency single frequency of 50 kHz, BIA 101, RJL Akern Bioresearch, Florence, Italy) [55], as previously reported [56–58]. The BIA analysis was performed according to the European Society of Parental and Enteral Nutrition (ESPEN) [40]. Participants were asked to remove their shoes and socks and the contact areas with electrodes (BIATRODES Akern Srl; Florence—Italy) were scrubbed with alcohol immediately before their placement on the hand and the ipsilateral foot, according to Kushner [59]. PhA was obtained as the relationship between the resistance (R) and reactance (Xc), according to the following formula: PhA (°, degrees) = Xc/R* (180/π). R is mainly dependent on tissue hydration, while Xc is associated with cellularity, cell size, and integrity of the cell membrane [60, 61]. BIA data were obtained under strictly standardized conditions; in particular, all participants had refrained from drinking, eating, and exercising for 6 h with no alcohol intake within 24 h before testing, were supine with limbs slightly spread apart from the body). The BIA-exam was performed by the same nutritionist and with the same device to avoid interobserver and interdevice variability. The BIA-tool was routinely checked with resistors and capacitors of known values, in particular the reliability for within-day and between-day measurements were < 1.6% for R, < 1.8% for Xc, and < 1.9% for R, < 2.1% for Xc, respectively. The coefficient of variation (CV) of repeated measurements of R and Xc at 50 kHz was assessed in 16 individuals (8 patients with acne and 8 controls): CVs were 1.4% for R and 1.5% for Xc.

### Adherence to the mediterranean diet

Adherence to the MD was assessed using the validated PREDIMED questionnaire, consisting of 14 items [62]. PREDIMED questionnaire was administered by a qualified nutritionist during a face-to-face interview and had already been used in previous studies [63–65]. PREDIMED score was calculated by assigning a score of one and zero for each item, thus obtaining a total score ranging from zero to 14 points. Based on the PREDIMED score obtained, the participants were classified into three PREDIMED categories, as follows: 0–5, low adherence to the MD; score 6–9, average adherence to the MD; and score ≥ 10, high adherence to the MD [62].

### Classification and severity assessment of acne

The dermatologists who evaluated the clinical severity of acne were blinded to the design of the study to prevent biases. Everyone had a complete dermatological examination including the GAGS, a quantitative scoring system to assess acne severity, first developed by Doshi et al. in 1997 [66]. The GAGS score is derived from the summation of six regional subscores. In detail, each point is derived by multiplying the factor for each region (the factor for chest and upper back is 3, for chin and nose is 1, and for forehead and each cheek is 2) by the most heavily weighted lesion within each region (4 for ≥ one nodule, 3 for ≥ one pustule, 2 for ≥ one papule, and 1 for ≥ one comedone). The regional factors were derived from the consideration of the density of pilosebaceous units and surface area and distribution. The severity was graded as three GAGS categories: patients with GAGS score 1–18 was defined as mild (where lesions include several noninflammatory comedones with less than inflammatory lesions); moderate GAGS score was from 19 to 30 (presence of many comedones, papules, pustules, but no nodules), and severe if GAGS score from 31 to 38 (presence of inflammatory nodules in addition to papules and pustules) [66, 67]. GAGS score was clinically evaluated by a single experienced dermatologist. To prevent rate biases, the dermatologists who evaluated the GAGS score were blinded to the design of the study.

### Statistical analysis

Results were expressed as mean ± SD and categorical variables are expressed as a percentage. The data distribution was evaluated by Kolmogorov–Smirnov test and the abnormal data (age, weight, PREDIMED score, and ECW) were normalized by logarithm. Skewed variables were back transformed for presentation in tables and figures. Differences between patients with acne and controls in body composition characteristics and PREDIMED scores were analyzed by Student’s paired *t*-test. The Chi-square (χ^2^) test was used to determine the significance of differences in the frequency distribution of dietary components included in the PREDIMED questionnaire and adherence to the MD. The differences among GAGS categories (mild, moderate, and severe acne) on age, anthropometric measurements, body composition characteristics, and PREDIMED score were analysed by between-group ANOVA test followed by the Bonferroni post hoc test. The correlations among GAGS with age, anthropometric measurements, BIA-parameters in patients with acne were assessed with the Pearson *r* correlation coefficients were estimated. Bivariate proportional odds ratio (OR) models, 95% interval confidence (IC), R^2^, *p*-value, were performed to assess of GAGS score on the 14 food items included in the PREDIMED questionnaire. Two multiple regression analysis models (stepwise method), expressed as R^2^, Beta (β) and *t*, with GAGS score as a dependent variable to estimate the predictive value of anthropometric measurements, BIA-parameters assessed, and adherence to the MD in patients with acne. Two-receiver operator characteristic (ROC) curves analysis was carried out to identify sensitivity and specificity, area under the curve (AUC), and CI, as well as cut-off values of PREDIMED score and PhA in detecting the predictive of the highest values of GAGS severity. Test AUC for ROC analysis was also calculated and we entered 0.747 for AUC ROC and 0.5 for null hypothesis values. An Alfa α level of 0.05 (type 1 error) and a β level of 0.2 (type II error) were used as the cut-off values for statistical significance. Variables with a variance inflation factor > 10 were excluded to avoid multicollinearity. Values ≤ 5% were considered statistically significant. Data were collected and analyzed using the MedCalc^®^ package (Version 12.3.0 1993- 2012-Mariakerke, Belgium).

## Results

In this case–control, a single center study evaluated 102 individuals; 51 patients with acne and 51 controls were matched for gender, age, and BMI. In particular, in patients with acne and control group, 38 (74.5%) subjects were females, age was 23.5 ± 5.9 *vs* 24.5 ± 3.9 years, *p* = 0.280; and BMI was 24.7 ± 4.1 vs 24.6 ± 1.7 kg/m^2^, *p* = 0.786. Of interest, patients with acne had the highest waist circumference values compared to their counterpart (79.1 ± 10.1 vs 73.1 ± 9.1 cm, *p* = 0.04). No differences were evident in lifestyle characteristics, particularly physical activity 12 subjects (23.5%) vs 11 individuals (21.6%); χ^2^ = 0.00, *p* = 1.00 and cigarette smoking habits 12 subjects (23.5%) vs 17 individuals (33.3%); χ^2^ = 0.77, *p* = 0.379; in patients with acne and control group, respectively.

In the patients with acne, the clinical severity of acne evaluated by GAGS was 22.08 ± 9.12, in particular, according to GAGS categories, 17 patients (33.3%) had mild acne, 26 patients (51.0%) showed moderate acne, and eight patients (15.7%) presented severe acne. No gender difference was evident in GAGS score (23.0 ± 10.2 vs 21.8 ± 8.9, *p* = 0.677; in males and females, respectively).

The anthropometric characteristics and body composition parameters of the study population evaluated by BIA, were summarized in Table 1. Significant difference values of all impedance parameters evaluated were found in patients with acne compared to controls. Of interest, patients with acne had the worst body composition parameters, in particular lower PhA (*p* = 0.003) and higher fat mass (*p* = 0.010) than controls.

**Table 1:** Body composition characteristics assessed by bioelectrical impedance analysis in patients with acne and control group

Parameters	Patients with acne n = 51	Control group n = 51	*p*-value*
Weight (kg)	68.4 ± 15.5	72.6 ± 4.1	0.059
R (Ω)	539.3 ± 78.3	424.6 ± 74.7	**< 0.001**
Xc (Ω)	55.8 ± 8.0	46.9 ± 8.4	**< 0.001**
PhA (°)	5.9 ± 0.6	6.3 ± 0.6	**0.003**
TBW (Lt)	36.5 ± 5.2	44.4 ± 5.1	**< 0.001**
ICW (Lt)	19.7 ± 2.8	24.7 ± 2.9	**< 0.001**
ECW (Lt)	16.9 ± 2.9	19.7 ± 2.5	**< 0.001**
ECW/ICW ratio	0.86 ± 0.10	0.80 ± 0.1	**0.003**
FM (Kg)	18.4 ± 12.0	13.4 ± 5.8	**0.010**
FFM (Kg)	49.9 ± 7.1	59.1 ± 5.5	**< 0.001**
BCM (Kg)	25.1 ± 5.2	32.7 ± 3.5	**< 0.001**

Significant difference values of all impedance parameters evaluated were found in patients with acne compared to controls. Results are expressed as mean ± SD. Weight and ECW were logarithmically normalized and transformed and back-transformed for presentation in table. Differences in the two groups were analysed by paired Student’s *t* test

*R* Resistance, *Xc* reactance, *PhA* phase angle, TBW total body water, *ICW* intra-cellular water, *ECW* extra-cellular water, *FM* fat mass, *FFM* free fat mass, *BCM* body cell mass

*A *p* value in bold type denotes a significant difference (*p* < 0.05)

Analyzing the frequency response of dietary items included in the PREDIMED questionnaire in detail, we found that the patients with acne consumed less vegetables (*p* = 0.026), fruits (*p* = 0.008), legumes (*p* = 0.041), fish (*p* < 0.001), and nuts (*p* = 0.003); and more soda drinks (*p* = 0.034), commercial sweets and confectionery (*p* = 0.021), as compared with control group (Table 2).

**Table 2:** Response frequency of dietary components included in the PREDIMED questionnaire in patients with acne and control group

Questions of PREDIMED questionnaire	Patients with acne		Control group				
	n	%	n		%	χ	*p*-value*
Use of extra virgin olive oil as main culinary lipid	48	94.1	44	86.3		0.99	0.318
Extra virgin olive oil > 4 tablespoons	42	82.4	42	82.4		0.07	0.795
Vegetables ≥ 2 servings/day	25	49.0	37	72.5		4.98	**0.026**
Fruits ≥ 3 servings/day	25	49.0	39	76.5		7.09	**0.008**
Red/processed meats < 1/day	37	72.5	27	52.9		3.39	0.065
Butter, cream, margarine < 1/day	39	76.5	45	88.2		1.69	0.194
Soda drinks < 1/day	29	56.9	40	78.4		4.48	**0.034**
Wine glasses ≥ 7/week	17	33.3	23	45.1		1.03	0.311
Legumes ≥ 3/week	33	64.7	43	84.3		4.18	**0.041**
Fish/seafood ≥ 3/week	23	45.1	45	88.2		19.46	**< 0.001**
Commercial sweets and confectionery ≤ 2/week	33	64.7	44	86.3		5.30	**0.021**
Tree nuts ≥ 3/week	22	43.1	38	74.5		9.11	**0.003**
Poultry more than red meats	36	70.6	45	88.2		3.84	0.051
Use of sofrito sauce ≥ 2/week	29	56.9	32	62.7		0.16	0.686

Patients with acne consumed less vegetables, fruits, legumes, fish, and nuts; and more soda drinks, commercial sweets, and confectionery, as compared with the control group. Results are expressed as numbers and percentage. Differences in the frequency response of dietary components included in the PREDIMED questionnaire were analysed by Chi-square (χ^2^) test

*PREDIMED* PREvención con DIetaMEDiterránea

*A *p* value in bold type denotes a significant difference (*p* < 0.05)

**Fig. 1:**
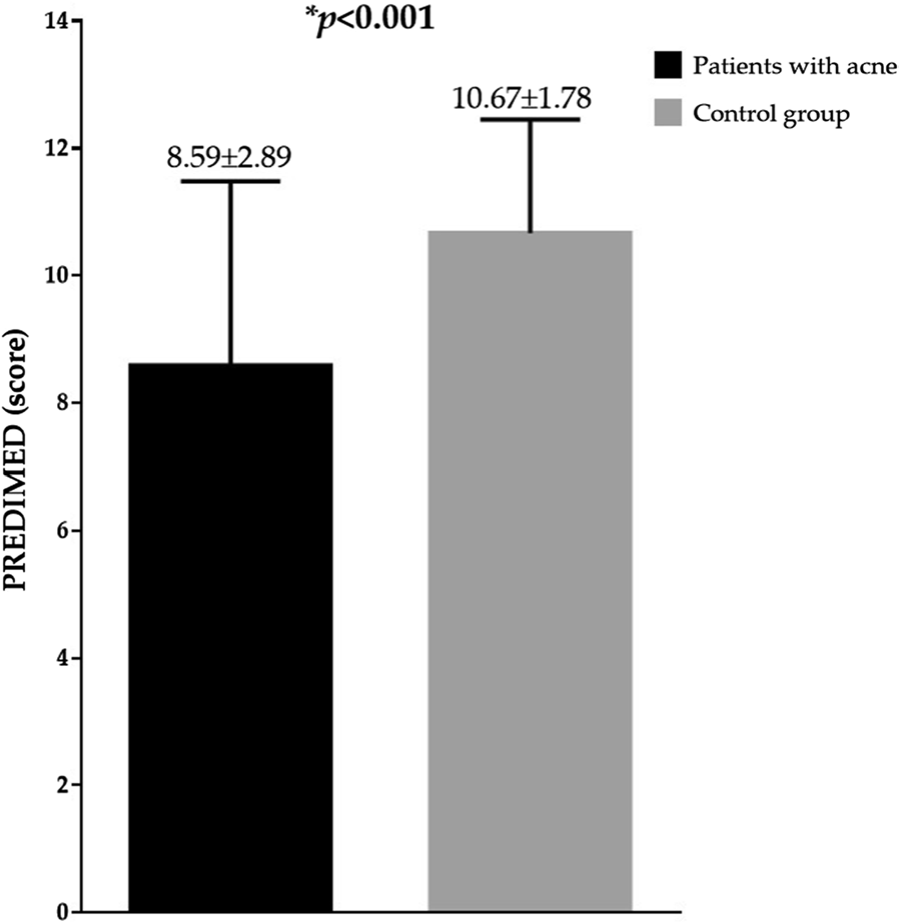
PREDIMED score in patients with acne and controls. Patients with acne presented the lowest adherence score to the Mediterranean diet compared to the control group. Difference in the PREDIMED score was analysed by paired Student’s *t* test. *A *p* value in bold type denotes a significant difference (*p* < 0.05). *PREDIMED* PREvención con DIetaMEDiterránea

Figure 1 reported PREDIMED scores in patients with acne and controls. As showed, patients with acne presented the lowest adherence score to the Mediterranean diet compared to the control group (*p* < 0.001). In detail, patients with acne compared to the control group, showed the highest percentage of low adherence to the Mediterranean diet (17.6% *vs* 0.0%; χ^2^ = 7.80, *p* = 0.005), and the lowest percentage of high adherence to the Mediterranean diet (43.1% vs 72.5%; χ^2^ = 7.88, *p* = 0.004); no difference was evident for the average adherence to the Mediterranean diet (39.2% *vs* 27.5%; χ^2^ = 1.10, *p* = 0.293).

Table 3 reports age, weight, BMI, waist circumference, and body composition characteristics assessed by BIA in patients with acne, across GAGS categories. In detail, stratifying the patients with acne according to GAGS categories, weight (*p* = 0.017), BMI (*p* = 0.002), waist circumference (*p* = 0.020), ECW (*p* = 0.050), ECW/ICW ratio (*p* = 0.007), and fat mass (FM) (*p* = 0.015) increased, while PhA decreased significantly (*p* = 0.006).

**Table 3:** Age, anthropometric characteristics, and body composition characteristics were assessed by bioelectrical impedance analysis in patients with acne, across GAGS categories

Parameters	Mild acne n = 17	Moderate acne n = 26	Severe acne n = 8	*p-value
Age (years)	24.1 ± 4.5	23.5 ± 6.2	22.3 ± 7.8	0.780
Weight (kg)	62.4 ± 5.9	68.6 ± 15.4	80.9 ± 22.8	**0.017**
BMI (kg/m^2^)	22.8 ± 1.5	24.8 ± 3.7	28.8 ± 5.9	**0.002**
Waist circumference (cm)	74.8 ± 4.9	79.6 ± 11.0	86.5 ± 11.3	**0.020**
R (Ω)	526.9 ± 73.6	548.3 ± 75.2	536.6 ± 102.6	0.684
Xc (Ω)	57.5 ± 8.5	56.2 ± 6.3	51.0 ± 11.1	0.163
PhA (°)	6.3 ± 0.6	5.9 ± 0.5	5.4 ± 0.6	**0.006**
TBW (Lt)	35.9 ± 3.9	36.2 ± 5.4	38.9 ± 6.9	0.386
ICW (Lt)	19.9 ± 2.6	19.5 ± 2.9	19.9 ± 3.0	0.867
ECW (Lt)	16.1 ± 1.8	16.8 ± 2.8	19.0 ± 4.1	**0.050**
ECW/ICW ratio	0.81 ± 0.08	0.86 ± 0.09	0.95 ± 0.11	**0.007**
FM (Kg)	13.2 ± 5.2	18.9 ± 10.6	22.8 ± 19.9	**0.015**
FFM (Kg)	49.1 ± 5.4	49.6 ± 7.3	53.1 ± 9.4	0.392
BCM (Kg)	26.6 ± 4.8	24.6 ± 5.2	23.3 ± 5.9	0.261

Stratifying patients with acne according to GAGS categories, weight, BMI, waist circumference, ECW, ECW/ICW ratio, and FM increased, while PhA decreased significantly. Results are expressed as mean ± SD. Weight and ECW were logarithmically normalized and transformed and back-transformed for presentation in table. Differences in the three groups were analysed by ANOVA test, with the Bonferroni test as post-hoc test. *A *p* value in bold type denotes a significant difference (*p* < 0.05)

*BMI* body mass index, *R* resistance, *Xc*, reactance, *PhA*, phase angle, *TBW* total body water, *ICW* intra-cellular water, *ECW* extra-cellular water, *FM* fat mass, *FFM* free fat mass, *BCM* body cell mass

**Fig. 2:**
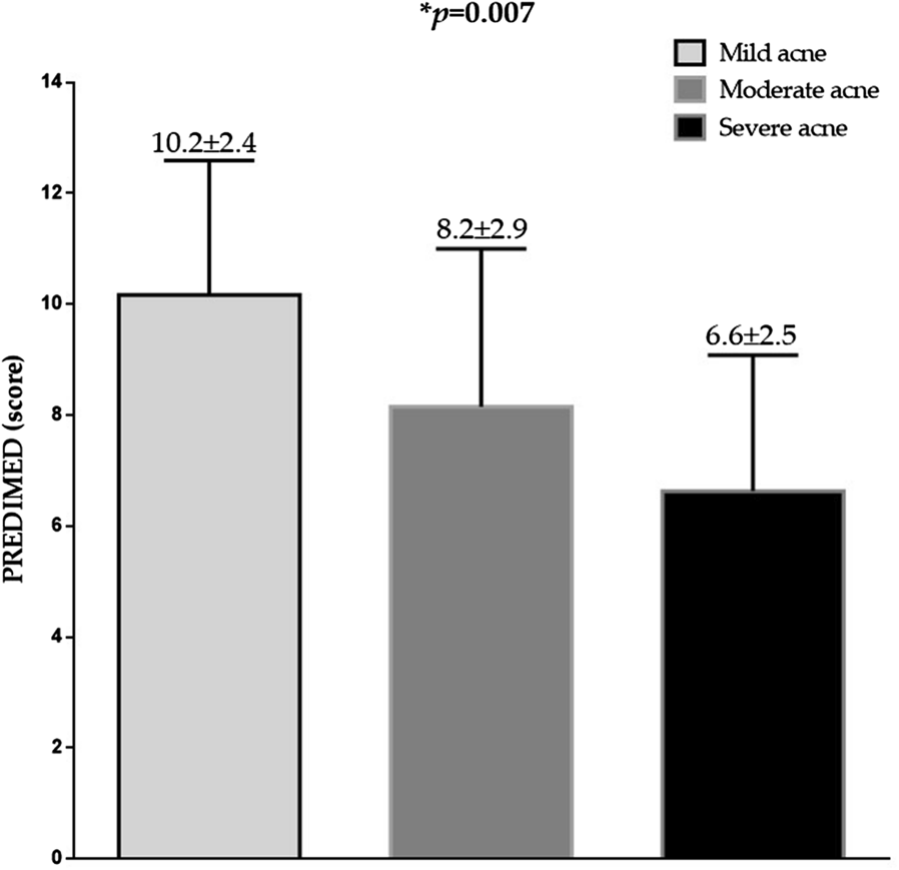
PREDIMED scores in patients with acne according to GAGS categories. PREDIMED score decreased significantly along as the clinical severity of acne increases. Differences in the three groups were analysed by ANOVA test, with the Bonferroni test as post-hoc test. *A *p* value in bold type denotes a significant difference (*p* < 0.05). *PREDIMED* PREvención con DIetaMEDiterránea

Likewise, dividing patients with acne according to GAGS categories, PREDIMED score decreased significantly along as the clinical severity of acne increases (*p* = 0.007), Fig. 2*.*

### Correlation analysis

Correlations among age, anthropometric measurements, and body composition characteristics with GASG score in patients with acne were reported in Table 4. Significant correlations were found between GAGS score, weight and waist circumference (*p* = 0.002), and BMI (*p* < 0.001). In addition, GAGS score was also negatively associated with Xc (*p* = 0.035) and PhA (*p* < 0.001), and positively correlated with ECW (*p* = 0.008), ECW/ICW (*p* = 0.001), and FM (*p* = 0.002); Table 4.

**Table 4:** Correlations among age, anthropometric measurements, and body composition parameters in patients with acne

Patients with acne (n = 51)			
Parameters	r	*p-value	
Age (years)	− 0.157	0.271	
Weight (kg)	0.432	**0.002**	
BMI (Kg/m^2^)	0.522	**< 0.001**	
Waist circumference (cm)	0.432	**0.002**	
R (Ω)	0.060	0.678	
Xc (Ω)	− 0.296	**0.035**	
PhA (°)	− 0.478	**< 0.001**	
TBW (Lt)	0.211	0.138	
ICW (Lt)	0.014	0.924	
ECW (Lt)	0.368	**0.008**	
ECW/ICW ratio	0.464	**0.001**	
FM (Kg)	0.427	**0.002**	
FFM (Kg)	0.220	0.122	
BCM (Kg)	− 0.233	0.100	

GAGS score was correlated with weight, waist circumference, BMI, Xc, PhA, ECW ECW/ICW, and FM. Weight and ECW were logarithmically normalized and transformed and back transformed for presentation in table. Correlations between variables were performed using Pearson *r* correlation coefficients

*BMI* body mass index, *R* resistance, *Xc* reactance, *PhA* phase angle, *TBW* total body water, *ICW* intra-cellular water, *ECW* extra-cellular water, *FM* fat mass, *FFM* free fat mass, *BCM* body cell mass

*A *p* value in bold type denotes a significant difference (*p* < 0.05)

Bivariate proportional OR models were performed to assess the association of GAGS scores with the 14 food items included in the PREDIMED questionnaire. In particular, in this model, the lowest GAGS scores were significantly associated with the highest OR of consumption Mediterranean food items, including the use and quantity of extra virgin olive (OR = 0.79, *p* = 0.020 and OR = 0.89, *p* = 0.025; respectively), fruits (OR = 0.88, *p* = 0.003), legumes (OR = 0.85, *p* = 0.001), and fish (OR = 0.84, *p* < 0.001); Table 5.

**Table 5:** Bivariate proportional OR model to assess the association between GAGS score and food items included in the PREDIMED questionnaire

	Patients with acne (n = 51)			
Questions	OR	*p* value	95% IC	R^2^
Use of extra virgin olive oil as main culinary lipid	0.79	**0.020**	0.63–1.01	0.12
Extra virgin olive oil > 4 tablespoons	0.89	**0.025**	0.81–0.99	0.11
Vegetables ≥ 2 servings/day	0.96	0.151	0.89–1.02	0.04
Fruits ≥ 3 servings/day	0.88	**0.003**	0.82–0.96	0.21
Red/processed meats < 1/day	0.96	0.206	0.89–1.03	0.03
Butter, cream, margarine < 1/day	0.98	0.634	0.91–1.06	0.01
Soda drinks < 1/day	1.00	0.993	0.94–1.06	0.01
Wine glasses ≥ 7/week	0.95	0.102	0.88–1.01	0.06
Legumes ≥ 3/week	0.85	**0.001**	0.76–0.94	0.27
Fish/seafood ≥ 3/week	0.84	**< 0.001**	0.75–0.93	0.32
Commercial sweets and confectionery ≤ 2/week	0.99	0.933	0.93–1.06	0.01
Tree nuts ≥ 3/week	0.95	0.134	0.89–1.02	0.05
Poultry more than red meats	1.02	0.515	0.96–1.09	0.01
Use of sofrito sauce ≥ 2/week	0.96	0.236	0.90–1.03	0.03

The lowest GAGS scores were significantly associated with the highest OR of consumption some Mediterranean food items, including the use and quantity of extra virgin olive, fruits, legumes, and fish. Bivariate proportional OR model, 95% IC, and R^2^

*GAGS* Global Acne Grading System, *PREDIMED* PREvención con DIeta MEDiterránea, *OR* odds ratio, *IC* interval confidence

*A *p* value in bold type denotes a significant difference (*p* < 0.05)

**Fig. 3:**
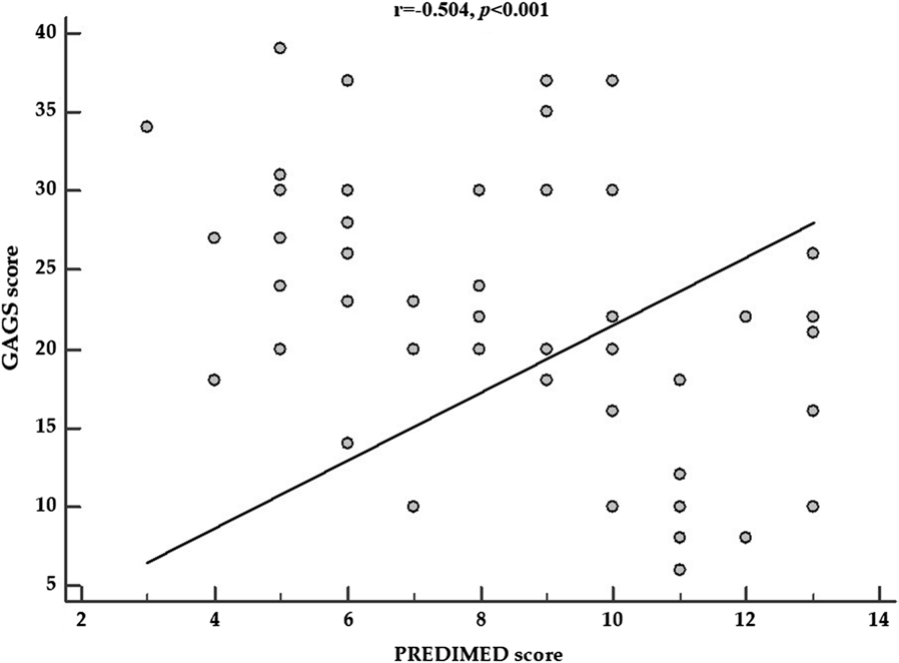
The correlation between GAGS score and PREDIMED score in patients with acne. GAGS score showed a negative association with PREDIMED score (r = − 0.504, *p* < 0.001) as shown in the figure. Correlations between two variables were performed using Pearson *r* correlation coefficients. *A p value in bold type denotes a significant difference (*p* < 0.05). *GAGS* global acne grading system, *PREDIMED* PREvención con DIeta MEDiterránea

Figure 3 reported the negative correlation between GAGS score and PREDIMED score (r = − 0.504, *p* < 0.001) in patients with acne.

To compare the relative predictive power of anthropometric measurements, BIA-parameters assessed by BIA, and adherence to the MD in patients with acne and associated with GAGS score, we performed two multiple regression analysis. The first model included anthropometric measurements, BIA-parameters, and PREDIMED scores. In this model, PREDIMED score was entered at the first step (*p* < 0.001), and the other variables were excluded from the analysis. Results were reported in Table 6. The second model included only BIA-parameters. In this model, PhA was entered at the first step (*p* < 0.001), and the other variables were excluded from the analysis; Table 6.

**Table 6:** Multiple regression analysis models (stepwise method) with the GAGS score as a dependent variable to estimate the predictive value of anthropometric measurements, BIA-parameters assessed, and adherence to the MD in patients with acne

Parameters	Multiple regression analysis			
	R^2^	β	t	*p* value*
Model 1				
PREDIMED score	0.239	− 0.504	− 4.09	**< 0.001**
Model 2				
PhA (°)	0.213	− 0.478	− 3.81	**< 0.001**

In the first model included anthropometric measurements, BIA-parameters, and PREDIMED score, PREDIMED score entered at the first step. In the second model including only BIA-parameters, PhA was entered at the first step

*GAGS* Global Acne Grading System, *PREDIMED* PREvención con DIeta MEDiterránea, *PhA* phase angle

*A *p* value in bold type denotes a significant difference (*p* < 0.05)

**Fig. 4:**
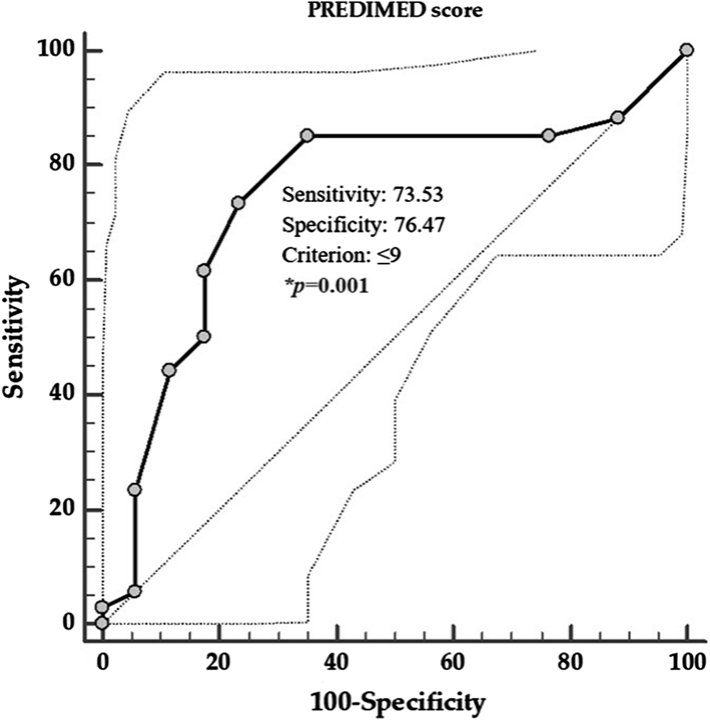
ROC for the value of PREDIMED score predictive of the highest GAGS severity. In the ROC analysis, the threshold value of PREDIMED scores predicting the highest GAGS severity were found at ≤ 9. *A *p* value in bold type denotes a significant difference (*p* < 0.05). *GAGS* global acne grading system, *PREDIMED* PREvención con DIeta MEDiterránea

**Fig. 5:**
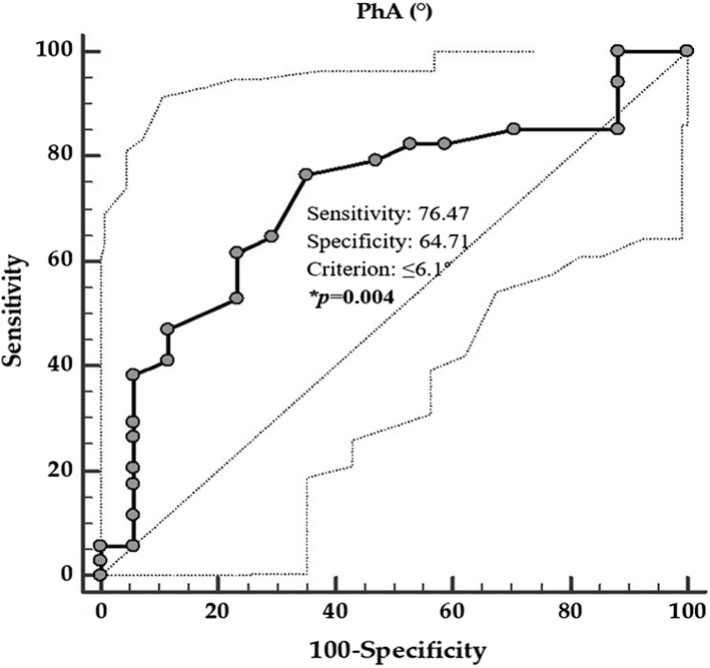
ROC for the value of PhA predictive highest GAGS severity. In the ROC analysis, the threshold value of PhA predicting the highest GAGS severity were found at ≤ 6.1°. *A *p* value in bold type denotes a significant difference (*p* < 0.05). *PhA* phase angle, *GAGS* global acne grading system

The first ROC analysis was performed to determine the cut-off values of PREDIMED score that was predictive of the highest value of GAGS severity (Fig. 4), while the second ROC analysis was performed to evaluate the cut-off value of PhA that was predictive of the highest value of GAGS severity (Fig. 5).

The threshold values of adherence to the MD predicting the highest GAGS severity were found at PREDIMED score ≤ 9 (*p* = 0.001, AUC 0.747, standard error 0.076, 95% CI 0.598 to 0.896; Fig. 4), in patients with acne. A value of PhA of ≤ 6.1° (*p* = 0.003, AUC 0.719, standard error 0.075, 95% CI 0.571 to 0.867) could serve as a threshold for a significantly increased risk of high value of GAGS severity.

## Discussion

In our study, patients with acne showed a lower adherence to the MD, smaller PhA, and higher waist circumference compared to the control group matched for gender, age, and BMI. In addition, the assessment of body composition by BIA showed a different water distribution in patients with acne compared to controls, in particular a lower total body water and body cell mass, and a higher ECW/ICW ratio and FM. The stratification of patients with acne in according to GAGS scores across GAGS categories allowed us to report a novel difference in PhA and PREDIMED scores across different GAGS categories with lower values in severe acne compared to moderate/mild acne. As expected, BMI and waist circumference were the highest in patients with acne with the highest grades of GAGS (severe). To date, to the best of our knowledge, this is the first study investigating nutritional status in the relationship between body composition and adherence to the MD in a sample of an adult population with acne across GAGS categories. Of interest, the most important result of the present study is the negative correlation between PhA and GAGS score. In the multiple regression analysis, PREDIMED score was the major determinant of anthropometric measurements, BIA-parameters, and adherence to the MD, and PhA was the major determinant of anthropometric measurements and BIA-parameters. Finally, based on the ROC curve analysis, the most sensitive and specific cut-offs for the PREDIMED score and PhA to predict the highest GAGS score were ≤ 9 and ≤ 6.1°, respectively. To date, this is the first study reporting the specific cut-offs of adherence to the MD and PhA which predict the clinical severity of acne.

PhA is an electrical parameter obtained directly from the BIA that has been used as an indicator of cell membrane function and as a marker of nutritional status in different populations [36]. In detail, whereas high PhA suggest a large number of intact cell membranes [36], low PhA are considered a marker for decreased cell membrane integrity or related to cell death [68]. Cell membrane integrity disarrangements, consequently of tissue injury, are a well-known condition related to the inflammatory status [69]. It is interesting to note how, in different clinical settings, including psoriasis [18, 70], hidradenitis suppurativa [20], obesity [71], and polycystic ovary syndrome [21], showed a strong relationship between PhA and inflammatory markers, including C-reactive protein [34, 72]. Our results demonstrated that in our group of patients with acne, an inflammatory skin disease, PhA values were significant lower unlike healthy subjects, matched for gender, age, and BMI, known cofactors that influence PhA. This let us to speculate that PhA, a known inflammatory parameter in several skin diseases [20, 70], could represent also a possible biomarker to quantify inflammation in subjects with acne, a chronic inflammation of the folliculo-pilosebaceous unit. Although in our study we have not evaluated specific inflammatory biomarkers, including C-reactive protein, this conclusion remains only speculative. Therefore, although our results cannot demonstrate a causal link, they suggest that PhA could be a marker of inflammation in patients with acne and PhA ≤ 6.1° predict the highest GAGS score.

Acne vulgaris is a common inflammatory disease of the pilosebaceous units of the skin, with a multifactorial and complex pathogenesis [1]. In a recent review on the role of diet on acne and its response to treatment, Baldwin H. et al., [17] found that in several evidence beyond the role of genetics, acne is considered due primarily to environmental factors and specific foods and dietary patterns have been included as possible triggers. Low-glycaemic index foods can influence the clinical severity of acne due to their positive influence on the reduction of seborrhea and keratinocytes turnover. Several evidence confirm the role of high-glycaemic index foods on the pathogenesis of acne. Hyperinsulinemia caused by the consumption of foods with a high glycemic index, increased insulin-like growth factor 1 levels, with consequent reduction of insulin-like growth factor-binding protein 3 and proliferation of basal keratinocytes thus leading to dysregulation of normal corneocytic apoptosis [73]. The first step in the formation of microcomedone is the acroinfundibular hyperkeratinization [74]. The main foods with a low glycemic index are legumes and fruit [75]. Our data report that acne patients consume fewer weekly servings of fruit and legumes than the non-acne group. Furthermore, the reduced consumption of these two Mediterranean foods were associated with worse clinical acne severity.

Moreover, foods high in omega 3 leading to a lower omega6/omega3 ratio due to their anti-inflammatory action are among the most possible causes of the reduction of the clinical severity of acne [76]. The higher omega6/omega3 ratio, characteristic of Western diet (poor in the consumption of fish, fresh vegetables, and legumes) is considered crucial in all inflammatory states, including [76, 77]. Of interest, in non-industrialized countries, the omega6/omega3 ratio is usually around 2–3:1, in reverse, in industrialized countries, such as USA, this ratio is close to 10:1; this could at least partially explain the difference in acne epidemiology [76, 78]. The main source of omega 3 in our diet is represented by the consumption of fish. Fish consumption has been reported to be associated with the clinical severity of acne [79]. In particular, Rietkerk and Woolf showed that patients with moderate and severe acne consumed fewer portions of fish than those with mild or no acne [80]. Likewise, Di Landro et al. [3], reported that fish consumption was associated with a protective effect on acne risk. Similar to these studies, our results demonstrated that patients with acne consumed fewer weeks fish servings than controls, and fish intake was negatively associated with the clinical severity of acne. The omega-3 fatty acids found in fish and the high fiber content in fruits and vegetables, could explain these observed protective effects from the consumption of these Mediterranean foods, which have been reported to lower the insulin-like growth factor 1 levels, thus reducing acne risk [17]. In addition, a high content of hormones active in the milk could act either on insulin levels leading to infundibular hyperkeratinization or that on pilosebaceous follicles (5-α-androstenedione and 5-α-pregnanedione), thus leading to worsening of acne [17, 81]. However, in the relationship between acne with single foods, it should be kept in mind that the diet is a complex combination of foods and nutrients. Thus, it is challenging to separate the effect of a single nutrient (eg omega 3, fiber, etc.) or food group (e.g. fish and legumes) from that of others in free-living populations [82], and it would therefore be more correct to evaluate the effects of an entire dietary pattern with respect to a single nutrient or food. In this context, beyond weight loss, a higher adherence to the MD has been reported to have a well-established anti-inflammatory activity, which is mainly due to the high intake of both polyunsaturated fatty acids omega 3 and antioxidants contained in extra-virgin olive oil, vegetables, fruits, and wine [83], and the microbiota-derived production of short chain fatty acids that are induced by dietary fiber [84].

However, only one study evaluated adherence to the MD with the clinical severity of acne [31]. In particular, in 2012, Skroza et al., in a community-based case–control study in 93 patients with acne and 200 controls affirmed that the MD score ≥ 6 revealed a protective effect towards acne [31]. This was the first study reporting a protective role of the MD in the pathogenesis of acne.

In our study population, the MD score was evaluated by PREDIMED score, which revealed that acne patients followed a dietary regimen farther from the MD than the one followed by healthy controls. Acne patients ate less Mediterranean foods than healthy controls. MD is richer in fruit, vegetables, and integral foods that could be protective for their both inflammatory and antioxidants properties [31]. The catalase and superoxide dismutase levels with increased oxidative stress, playing an important role in the pathogenesis of acne [85]. MD is a well-established health-promoting dietary pattern. There is evidence that high adherence to the MD is negatively correlated with insulin resistance [86], adiposity [87], inflammation and immune system function [28, 29], gut microbiota [88], and endocrine dysfunction [89], all conditions associated with acne [17]. Although our results cannot demonstrate a causal link, they suggest that high adherence to the MD could be a marker of inflammation in patients with acne and the PREDIMED score ≤ 9 predicted the highest GAGS score. On this basis, it is conceivable that a high adherence to the MD might be considered one of the best nutritional strategies for the management of patients with acne.

Some limitations should be reported in the present study. First, being a cross-sectional study, the cause-effect association between PhA, PREDIMED score and clinical severity of acne cannot be determined. Likewise, the design of this study did not draw a final conclusion on the prognostic value of PhA and degree of adherence to the MD in the prediction of clinical severity of acne.

Second, the possible underlying inflammatory linking PhA and PREDIMED score with the clinical severity of acne should be better investigated by measuring serum inflammatory biomarkers, such as c-reactive protein levels and IL-6; thus, our hypothesis of a beneficial effect of both high PhA and high adherence to the MD on the clinical severity of acne remains largely speculative. However, we point out that this was the first study reporting in patients with acne the negative correlation among PhA values and PREDIMED score with clinical severity of acne.

Third, the sample size was relatively small. Nevertheless, we have calculated the sample size calculated by the differences of means of PREDIMED score in patients with acne and controls, using 95% statistical power. Furthermore, patients and controls shared the same geographical area, being a monocentric study, therefore both groups had likely similar food availability and dietary consumption patterns, and this allowed to improve the homogeneity of the study population.

Fourth, the proposed cut-off points of PhA and PREDIMED score for identifying the highest clinical severity of acne should be used with caution until an appropriate cross-validation will be performed and the data of clinical evidence in larger population samples will be made available.

A strong point of our research with the aim of minimizing interoperator variability is that only the very same expert nutritionist performed and interpreted BIA-parameters, anthropometric measurements, and PREDIMED questionnaires. In addition, the diagnosis of acne that was clinically evaluated by dermatologists and not self-reported.

An additional strong point is that we included only naïve treatment patients and both acne patients and matched controls have been well characterized.

In addition, although we based the statistical analysis and results mostly on raw BIA measurements (as PhA) rather than BIA volumetric parameters, the evaluation of FM, FFM, and body cell mass were not validated by gold standard reference methods, such as dual-X ray absorptiometry. For this reason, it is essential that expert nutritionists are mandatory for execution and especially for the interpretation of BIA measurements, in particular PhA.

Finally, adherence to the MD has been adequately assessed by the PREDIMED questionnaire, the gold standard among adherence to the MD questionnaires [90]. PREDIMED questionnaire, less time-demanding of compilation, is less expensive, and requires less collaboration from subjects than other more comprehensive methods, such as the full-length food questionnaire [62]. Of interest, PREDIMED questionnaire allows one to provide feedback to the subjects immediately after the interview is completed. Moreover, to minimize any bias related to the filling of the questionnaire and to avoid the interoperator variability, PREDIMED questionnaire was face-to-face administered and not self-reported.

## Conclusion

In summary, in the present cross-sectional, case–control, observational study, we reported for the first time: (i) smaller PhAs and low adherence to the MD in patients with acne compared to the control group matched for gender, age and BMI; (ii) direct associations among PhA and PREDIMED score with clinical severity of acne evaluated with GAGS score, suggesting the use of both PhA and PREDIMED score as a simple and inexpensive markers for both the clinical severity of acne and chronic inflammation. In addition, these data could support a therapeutic role of the Mediterranean dietary pattern in patients with acne, by contributing to reduce the inflammatory status that paves the way for an increase of clinical severity of acne. As possible translational applications, these results suggest that specific cut-off values for the PhA and PREDIMED score, contributing to identify patients with higher clinical severity of acne and could therefore be included as an auxiliary tool in the complex dermatological evaluation of the clinical severity of acne, and identifying those patients who could get an additional benefit from careful dietary interventions. In conclusion, our findings highlight the importance of nutritional assessment by a qualified nutritionist in patients with acne, not only for providing nutritional advices but also to identify subjects in which further performing clinical assessments.

## Data Availability

All data generated or analyzed during this study are included in this published article.

## Abbreviations

PhA: Phase angle
BIA: Bioelectrical impedance analysis
MD: Mediterranean diet
PREDIMED: PREvención con DIeta MEDiterránea
GAGS: Global acne grading system
ECW: Extracellular water
ICW: Intracellular water
SD: Standard deviation
R: Resistance
Xc: Reactance
CV: Coefficient of variation
OR: Odds ratio
IC: Interval confidence
ROC: Receiver operator characteristic
AUC: Area under the curve
